# Childhood Sustained Hypercalcemia: A Diagnostic Challenge

**DOI:** 10.4274/jcrpe.4247

**Published:** 2017-12-15

**Authors:** Nisa Eda Çullas İlarslan, Zeynep Şıklar, Merih Berberoğlu

**Affiliations:** 1 Ankara University Faculty of Medicine, Department of Pediatrics, Ankara, Turkey; 2 Ankara University Faculty of Medicine, Department of Pediatric Endocrinology, Ankara, Turkey

**Keywords:** Hypercalcemia, hyperparathyroidism, idiopathic infantile hypercalcemia, malignancy-related hypercalcemia, pediatrics

## Abstract

**Objective:**

This study aimed to call attention to hypercalcemia, a rare finding in children which carries the potential of leading to serious complications without proper intervention.

**Methods:**

Diagnosis, treatment, and clinical course of children with sustained hypercalcemia admitted between the years 2006-2016 were reviewed. Group 1 [parathyroid hormone (PTH)-dependent] consisted of patients with high/unsuppressed PTH levels and group 2 (PTH-independent) included cases with normal/suppressed PTH levels.

**Results:**

Twenty patients (11 male, 9 female) with a median age of 6.25 (0.03-17.88) years were evaluated. Symptoms were mostly related with the gastrointestinal system, while six patients (30%) were asymptomatic. Physical examination findings were diverse, non-specific, and normal in four patients (20%). Median time of diagnosis was 45 (2-720) days. Patients were divided into group 1 (n=7) and group 2 (n=13). Most frequent etiologies were primary hyperparathyroidism (n=5), idiopathic infantile hypercalcemia (IIH) (n=5), and malignancy (n=4). A moderate positive correlation was noted between serum calcium and creatinine levels (r=0.53, p=0.02). Nephrocalcinosis was the most common complication (n=9) (45%). Treatment was not implemented in 2 patients with mild hypercalcemia, while other patients received medical treatment ± surgery. Treatment-resistant patients were cases of malignancies and neonatal severe hyperparathyroidism. Long-term follow-up displayed resistant hypercalciuria in three infants diagnosed as IIH.

**Conclusion:**

Many patients with childhood hypercalcemia are asymptomatic or exhibit a non-specific and heterogeneous clinical presentation, resulting in delayed diagnosis. Mild cases may not be recognized, while symptoms may be missed in the presence of accompanying illnesses. Nevertheless, serious complications may only be avoided with prompt diagnosis and intervention.

**What is already known on this topic?**

Hypercalcemia is a rare finding in children which may be due to a wide variety of factors and possibly left undiagnosed in mild elevations, while carrying the potential to lead to serious complications if proper intervention is not achieved.

**What this study adds?**

Suspicion of hypercalcemia and measurement of serum calcium level in children with clinical findings not attributable to any other clinical condition is essential. In case of hypercalcemia, young infants are more vulnerable to development of nephrolithiasis and should be evaluated as soon as possible.

## INTRODUCTION

Hypercalcemia is defined as a state of total serum calcium level consistently higher than 11 mg/dL (2.75 mmol/L) or an ionized calcium concentration exceeding 5.4 mg/dL (1.35 mmol/L) (1). Measurement of ionized calcium is recommended for correct interpretation of hypercalcemia as it represents the physiologically active fraction. If not available, total calcium levels may be taken into account, while “corrected calcium” levels should be calculated in cases of hypoalbuminemia (2).

Hypercalcemia is a commonly encountered problem in adults, with most cases due to hyperparathyroidism and malignancy. In children, although not frequent, hypercalcemia may be observed in a wide range of diseases. Its clinical features tend to be diverse, non-specific, and sometimes insignificant. Some patients may be asymptomatic, while hypercalcemia can cause serious end-organ damage such as neurological complications and kidney failure if not diagnosed and treated promptly (3,4,5).

Understanding the physiology of calcium balance is mandatory for correct diagnosis and treatment. Calcium balance is maintained by complex interplay between the parathyroid gland, bones, the intestine, and the kidneys (6). The principle regulators of this process are considered to be parathyroid hormone (PTH) and 1,25-dihyroxyvitamin D [1,25-(OH)_2-_D_3_] (3). The calcium-sensing receptor (CaSR), which is expressed mainly in the parathyroid gland and kidneys, plays a critical role in the sustainment of plasma calcium levels. Increased ionized calcium levels activate CaSR which in turn acts to reduce PTH secretion (3,4). PTH is secreted at a rate inversely proportional to the circulating level of ionized calcium (7). It functions to mobilise calcium from bones through stimulation of osteoclastic activity. PTH also endorses reabsorption of calcium while inhibiting renal phosphate reabsorption at the distal renal tubule and enhances 1-α hydroxylation of 25-hydroxyvitamin D_3_ (25-OH-D_3_) to 1,25-(OH)_2-_D_3_ (calcitriol). Calcitriol promotes calcium and phosphate absorption from the intestine, increases bone mineralization and renal calcium reabsorption.

Identification of the underlying pathology of hypercalcemia is essential as treatment modality should be targeted on the specific cause. It is practical to recognize circulating levels of PTH initially and decide whether the cause of hypercalcemia is PTH-dependent or PTH-independent (4).

This study aimed to call attention to hypercalcemia, a rare finding in children, which may be due to a wide variety of factors and possibly left undiagnosed in mild elevations, while carrying the potential to lead to serious complications if proper intervention is not achieved.

## METHODS

This retrospective cohort study was conducted in Ankara University Faculty of Medicine Department of Pediatric Endocrinology. The study protocol was approved by the Clinical Research Ethics Committee of our university (approval number: 10-428-16; May 2016).

We identified a total of 20 children who were diagnosed to have “sustained” hypercalcemia in the time period between 2006 and 2016. Sustained hypercalcemia is defined as persisting hypercalcemia detected in at least two consecutive days of measurement (8). Exclusion criteria were outlined as incomplete data and “transient” hypercalcemia, which is characterized as normal serum calcium measurement following a single high value.

Each patient’s age, presenting symptoms, vital signs (with particular attention on pulse rate and blood pressure), and physical examination findings related with hypercalcemia were recorded.

### Laboratory Evaluation of Patients

Laboratory evaluations performed to establish the underlying diagnosis were noted for each patient. Corrected total serum calcium, phosphate, alkaline phosphatase, PTH, electrolytes, renal function tests, 25-OHD, and urinary calcium/creatinine ratio results at the time of diagnosis and prior to treatment were recorded. Further investigations [parathormone-related peptide (PTHrP), genetic analysis, parathyroid gland ultrasonography, Tc-99m sestamibi scanning of the parathyroid gland, and examination of parents for abnormalities of calcium homeostasis] were likewise documented in selected patients.

### Grouping of Patients

Definite diagnosis indicating the underlying etiology of hypercalcemia for each patient was listed. Finally, patients were categorized into two groups based on PTH level. Group 1, the PTH-dependent group, consisted of patients with high/unsuppressed PTH levels. Group 2 included cases with normal/suppressed PTH levels and was called the PTH-independent group. We defined PTH suppression as a PTH level of <14 pg/mL (normal range: 14-72 pg/mL) (9). Hypercalcemia was defined as mild, moderate, or severe according to corrected total serum calcium levels. We considered total serum calcium levels between 11-12 mg/dL as mild, >12 and ≤14 mg/dL as moderate, and levels higher than 14 mg/dL as severe hypercalcemia (10).

### Evaluation of Complications

Urinary tract ultrasonography was performed to detect possible nephrolithiasis and/or nephrocalcinosis resulting from hypercalciuria. Skeletal survey findings were recorded in patients who had undergone the evaluation. Hypertension, band keratopathy, and acute pancreatitis were reported as well.

### Treatment and Long-term Follow-up of Patients

Treatment modalities received by each patient for hypercalcemia, as well as the period of time required to normalize serum calcium levels and the course of the hypercalcemia in long-term monitoring were recorded.

### Statistical Analysis

Statistical analysis was performed using the SPSS statistical package (v.21.0). Counts and percentages for categorical variables, median values and ranges for continuous variables were recorded. Count comparisons were analyzed using non-parametric methods due to the small size of the study group. Contingency tables (2x2) were analyzed using Fisher’s exact test, while higher dimensional tables were analyzed using the chi-square test. Mann-Whitney U and Kruskal-Wallis tests were conducted for comparisons involving continuous variables. Degree of association between variables was evaluated by Spearman’s correlation coefficient. A p-value of less than 0.05 was considered statistically significant.

## RESULTS

A total of 20 patients (9 female, 11 male) with a median age of 6.25 (0.03-17.88) years were evaluated. The most frequent presenting symptoms were related with the gastrointestinal system (nausea, vomiting, anorexia, abdominal pain, constipation), while 30% (n=6) of patients did not show any symptoms related with hypercalcemia. Of these, 4 cases were asymptomatic and were diagnosed incidentally, while two patients had malignancy and displayed symptoms associated with the primary diagnosis. In addition, we observed a wide range of symptoms such as polyuria, polydipsia, change in behavior, weight loss, muscle weakness, bone pain, and restlessness (Table 1). The median time elapsed from the onset of symptoms until diagnosis was 45 (2-720) days in symptomatic patients. With exception of a history of nephrolithiasis in first-degree relatives in two patients, family histories did not reveal presence of hypercalcemia, parathyroid gland disease and/or surgery.

We encountered diverse and non-specific physical examination findings such as signs of dehydration, hypertension, syndromic features, short stature, proximal myopathy, acute pancreatitis, and findings suggestive of malignancy such as lymphadenopathy and hepatosplenomegaly. Physical examination was recorded as normal in four patients (20%).

Median corrected total serum calcium level of the study group at the time of diagnosis was 12.6 (11-16.5) mg/dL. Excluding the patient with chronic renal insufficiency, a moderate positive correlation was noted between corrected total serum calcium and creatinine levels (r=0.53, p=0.02) although serum creatinine level was within the normal range in all patients.

**Figure 1:**
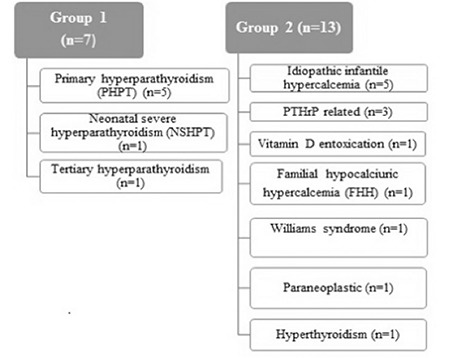
Etiology of hypercalcemia PHPT: primary hyperparathyroidism, NSHPT: neonatal severe hyperparathyroidism, PTHrP: parathormone-related peptide, FHH: familial hypocalciuric hypercalcemia

Based on PTH level, group 1 (n=7) consisted of patients with elevated or unexpectantly unsuppressed PTH measurement (Figure 1). Group 2 (n=13) comprised patients with normal or suppressed PTH level. The most frequent etiologies were primary hyperparathyroidism (PHPT) (n=5), idiopathic infantile hypercalcemia (IIH) (n=5), and malignancy-related hypercalcemia (n=4).

Median PTH level in group 1 patients was 282.6 (10-764) pg/mL. Median age was 16.32 (10.02-17.25) years. Six patients in this group had high PTH levels. One adolescent patient with bone cysts exhibited a normal PTH level. Parathyroid gland ultrasonography in this patient revealed a parathyroid adenoma. Parathyroid ultrasonography revealed hypoechoic, lobulated extrathyroidal masses with well-defined margins in 6 patients. Possible diagnosis was parathyroid adenoma. Five of these patients were diagnosed as PHPT. One patient with chronic renal insufficiency was considered to develop tertiary hyperparathyroidism following renal transplantation. Only one patient, who was a neonate, displayed a high PTH level and normal ultrasonographic findings. Magnetic resonance imaging of the neck was likewise normal in this patient. This same patient was found to have a de novo homozygote CaSR gene mutation (PI1e81Lys point mutation) and was diagnosed as neonatal severe hyperparathyroidism (NSHPT) (11). His parents were heterozygote for the same mutation. Tc-99m sestamibi scanning of the parathyroid gland was conducted in five patients reporting focal increased activity compatible with parathyroid adenoma in three. Operative and pathologic findings revealed a single adenoma in six patients, while two parathyroid glands were reported to be hyperplastic in the patient with NSHPT.

In group 2, five infants of ages ranging between 15 days to 8 months had presented with mild hypercalcemia, low serum PTH, normal serum 25OHD, and increased urinary calcium/creatinine ratio. These patients were accepted as cases of IIH. Mutational screening of CYP24A1 gene accomplished in one patient revealed a normal configuration.

Three patients with malignancies (chronic myeloid leukemia, hepatoblastoma, and rhabdomyosarcoma) in this group had elevated PTHrP (13.3-55.1 pg/mL) and low PTH levels. One patient with acute lymphoblastic leukemia displayed moderate hypercalcemia, low serum PTH (10 pg/mL), normal serum 25OHD, PTHrP, and renal function tests.

One female patient who exhibited mild hypercalcemia, low PTH (8.6 pg/mL), normal 25OHD, and hypocalciuria was found to carry a de novo heterozygote mutation of the CaSR gene (p.994delK (C.2981del AGA) and was diagnosed as a case of familial hypocalciuric hypercalcemia (FHH). Her mother was heterozygote for the same mutation, was normocalcemic, and had a normal urinary calcium/creatinine ratio.

An eighteen-month-old infant with moderate hypercalcemia and distinctive facial features together with supravalvular aortic stenosis was noted to have an elastin gene mutation on chromosome 7q.11.23 in fluorescent in situ hybridization analysis. This patient was diagnosed as Williams syndrome.

One child recently diagnosed as hyperthyroidism was found to have mild hypercalcemia which was detected incidentally. This four-month-old baby was referred because of restlessness, anorexia, and vomiting. She had severe hypercalcemia (16.4 mg/dL). She was admitted 10 days following the last dose of oral vitamin D (300,000 IU/dose) which she had received for 3 consecutive months. Serum 25OHD was high (291.4 mcg/L), serum PTH was low (5.2 pg/mL) in this patient and she was hypercalciuric. Vitamin D intoxication was the final diagnosis.

Bone mineral densitometry of two patients revealed results close to lower limits of normal (Z score: -1.75 and -1.8). One patient with parathyroid adenoma was found to have osteitis fibrosa cystica, detected by both skeletal survey and computed tomography. Band keratopathy was not recognized in any patient. All patients were evaluated with ultrasonography for renal stones. Medullary nephrocalcinosis and/or nephrolithiasis were identified at the time of diagnosis in seven patients, while two patients developed renal stones during follow-up, reaching a frequency of 45% in the total study group. The median age of patients with renal stones was younger compared to patients without renal stones (p=0.03), while no relationship was detected between serum total calcium, serum PTH level, urinary calcium/creatinine level, and presence of nephrocalcinosis and/or nephrolithiasis (Table 2).

Hypertension was detected in two patients. Two patients (PHPT) were diagnosed as acute pancreatitis with hypercalcemia. One of these patients had been priorly diagnosed as Familial Mediterranean fever (FMF) with no response to colchicine treatment.

### Therapeutic Interventions and Clinical Course

Two patients with PHPT and mild hypercalcemia did not receive medical therapy before surgery. Intravenous hydration and bisphosphonates were applied to the remaining patients in group 1. Normocalcemia was achieved in two patients before surgery and on the postoperative day one in the remaining patients, with no recurrence. Although the neonate diagnosed as NSHPT with severe hyperalcemia received intensive treatment modalities including cinacalcet, only partial response was accomplished before surgery. When surgery took place at seven months, three parathyroid glands were resected and total thyroidectomy was performed because of inability to locate the fourth parathyroid gland. Normocalcemia was maintained on the first postoperative day, but serum calcium level reached 17.5 mg/dL in one week.

Two patients (IIH and FHH) resembling mild hypercalcemia were not given any treatment and spontaneous resolution was observed in both. The remaining patients with IIH were treated with glucocorticoids ± intravenous hydration. Resolution of hypercalcemia took 4-14 days. Repeated mild hypercalcemia was identified in 2 patients. Four infants with IIH were followed for 4-6.5 years and transient hypercalciuria was achieved in three of them.

Intravenous hydration and bisphosphonates were administered to patients with malignancy. Hypercalcemia resolved in 7-22 days but reappeared in all. Intravenous hydration, furosemide, glucocorticoids, and bisphosphonates were administered to the infant with vitamin D intoxication. Normocalcemia was achieved in 15 days with no recurrence. The patient with Williams syndrome was treated with intravenous hydration, furosemide, and glucocorticoids. Hypercalcemia ameliorated within two days. The patient diagnosed as Graves’ disease was treated with antithyroid drugs with achievement of normocalcemia in four days. He was subsequently operated.

## DISCUSSION

Hypercalcemia is an infrequent finding in children but may be observed in a diverse range of clinical conditions with non-specific signs and symptoms in many cases. Moreover, determination of serum calcium is not included in many routine biochemistry profiles in children. Consequently, recognition of hypercalcemia can be a challenge in many occasions. Our study attempted to call an attention to this electrolyte imbalance.

Previous reviews mention diverse, sometimes non-specific, and occasionally absent clinical presentation in childhood hypercalcemia (3,5). We encountered similar results in our study, indicating the importance of suspecting hypercalcemia in patients with clinical findings non-attributable to other clinical conditions.

We found the median time of diagnosis as 45 days from the initiation of symptom. This period was even longer in some cases. For instance, two patients previously diagnosed as FMF and bone cysts were subsequently diagnosed as PHPT 36 and 72 months later, respectively. We suppose the main reason for delayed diagnosis was presence of non-specific and diverse clinical findings and symptoms. Moreover, a study of patients with PHPT reported the median time of diagnosis as 24 months (range: 1-60 months) which signified even later detection than in our cohort (12).

We noted a moderate positive correlation between total serum calcium and creatinine levels. We argue that normal but close to upper limits of serum creatinine levels might serve as an early indicator of renal damage in hypercalcemia.

A practical approach to hypercalcemia in children requires measurement of accompanying levels of PTH to determine whether the cause is PTH-dependent or PTH-independent (4). Unlike adults, PHPT accounts for only a small portion of childhood hypercalcemia and represents 1% of all cases (12,13). On the other hand, we defined 7 cases of PTH-dependent hypercalcemia, constituting 35% of our series. We think that this high ratio resulted from our institution serving as a reference tertiary center for complex cases necessitating multidisciplinary approach including surgery. Consistent with previously published reports, the majority of this group were adolescents (5,13). The hallmark laboratory findings of PHPT are hypercalcemia in the setting of elevated or inappropriately normal serum PTH level (14). One study including 52 children with PHPT reported that 85% of patients displayed elevated and 15% normal PTH (13). We had one patient with a normal PTH level. As in our case, we suggest further evaluation (e.g. parathyroid gland imaging) of children, especially adolescents, with unexplained hypercalcemia despite a normal PTH level.

Imaging to define the source of hyperparathyroidism comprise renal and neck ultrasonography and Tc-99m sestamibi scanning of the parathyroid gland. These imaging modalities achieve to localize adenomas in 80-90% of older children but their diagnostic success is lower in multigland hyperplasia (3). We observed positive findings in three of five patients who had undergone parathyroid gland scanning. Ultrasonographic evaluation indicated parathyroid adenoma in six patients, while it failed to detect any abnormality in one neonate. This is compatible with the literature informing the efficacy of ultrasonography in detection of parathyroid gland abnormality in only a third of cases with NSHPT (2).

IIH is a rare cause of childhood hypercalcemia, mostly appearing in the first year of life (15,16). Serum PTH level is suppressed with normal or elevated 25OHD and calcitriol levels. It was first identified in the 1950s in the UK when nearly 200 infants with hypercalcemia of unknown origin were reported in a short period of time (17). Excluding infants carrying dismorphic features and supravalvular aortic stenosis who were soon diagnosed as Williams-Beuren syndrome, most infants were regarded as “idiopathic”. Henceforth, IIH was considered as a diagnosis of exclusion in infants with hypercalcemia (1). In 2011, Schlingmann et al (18) were the first researchers who defined loss-of-function mutations in the cytochrome P450 24A1 gene (CYP24A1), which encodes vitamin D hydroxylase in patients with IIH. Mutations of this metabolising enzyme of calcitriol causes increased sensitivity to vitamin D. Another gene defect involving SLC34A1 gene which encodes renal sodium-phosphate co-transporter was identified later in patients with IIH (19). Five infants of our study group without dismorphic features displayed mild hypercalcemia, hypercalciuria, low serum PTH, normal serum 25OHD, and were diagnosed as IIH. We performed mutational screening of CYP24A1 gene in only one of our patients which reflected a negative result. The rest of our patients were diagnosed before description of this gene.

Unlike adults, hypercalcemia of malignancy is rare in children with an overall incidence of 0.4-1.3% (20). We had four patients with malignancy. Three of them were considered to develop humoral hypercalcemia (high PTHrP level), whereas one patient resembling normal PTHrP was believed to have hypercalcemia related with tumor synthesis of osteoclast-activating factors [interleukin (IL)-1, IL-6, tumor necrosis factor-α, and prostaglandins] (3,21).

Vitamin D intoxication is another cause of childhood hypercalcemia. Although the specific vitamin D intake that causes excess or intoxication is not clearly defined in pediatrics, current recommendations address 4000 IU as the upper tolerable daily intake (22). Furthermore, polymorphisms in genes that function in regulation of vitamin D synthesis have been implicated to affect circulating vitamin D levels (23). The Endocrine Society defines serum concentrations of 25OHD exceeding 150 ng/dL as intoxication for both children and adults and this cut-off is accepted by the Pediatric Endocrinology Society (24,25). One patient of our study group presenting with severe hypercalcemia was diagnosed as vitamin D intoxication.

FHH is an autosomal dominant disorder and typically carries good prognosis not necessitating treatment and close monitoring (2). Most patients carry a heterozygous inactivating mutation in the CASR gene, leading to an elevation of the set point for maintaining normal plasma calcium levels (26). Although it is characterised by a positive family history, hypercalcemia and hypocalciuria accompanied by normal or marginally elevated PTH, some patients may not demonstrate all these features (27). In such instances, biochemical profiles may hardly be distinguished from that of PHTP (3). Thus, molecular screening for CASR mutations should be performed when FHH is considered in the differential diagnosis of hypercalcemia, especially in the absence of one or more cardinal features (9). One of our patients demonstrated the aforementioned characteristics with the exception of a normal PTH level and a family history of hypercalcemia. Finally, both the patient and her mother were found to carry the same heterozygote CASR mutation.

Williams syndrome (Online Mendelian Inheritance in Man 194050) is caused by a deletion in chromosome 7 presenting with typical facial features and congenital heart disease, most commonly supravalvular aortic stenosis and peripheral pulmonary stenosis. Approximately 15% of cases are associated with hypercalcemia during infancy which is believed to result from increased sensitivity to vitamin D (7,28). One of our patients exhibited typical features of Williams syndrome along with severe hypercalcemia and this diagnosis was verified by genetic analysis.

It is known that mild hypercalcemia resulting from stimulation of osteoclastic bone resorption by high plasma levels of T3 may occur in hyperthyroidism (3). We had a similar patient who showed resolution of hypercalcemia after restoring normal thyroid gland functions.

We observed that nephrocalcinosis and/or nephrolithiasis was mostly present at younger ages, especially occurring in the first year of life. We believe this finding may indicate that younger children with hypercalcemia are more vulnerable to occurrence of nephrolithiasis and should be evaluated for this complication as early as possible.

In our series, resolution of hypercalcemia was achieved in all patients with PHPT and one patient with tertiary HPT following surgery without recurrence. A study consisting of 52 study subjects reported repeated operations in 17% (n=9) of patients with PHPT (12). Although our data indicate better surgical success and less recurrence of adenomas, we recognize that the follow-up period of our patients [median 11 (6-54) months vs. 13 (3-23] years) was relatively short as compared to the study cited above.

We had one neonate who presented with a severe course of hypercalcemia consistent with previous reports of NSHPT and repeated hypercalcemia occurring shortly after surgery, suggesting an ectopic fifth parathyroid gland (29,30).

One out of five patients diagnosed as IIH did not require treatment, while repeated hypercalcemia was observed in two cases. Studies suggest that although hypercalcemia usually resolves spontaneously in months in IIH, hypercalciuria may persist for years. A clinical study investigating long-term follow-up of infants diagnosed as IIH reported 63% resolution of hypercalcemia by 2 years of age, while persistent or recurrent hypercalciuria was identified in 5 out of 11 patients (15). Our experience reflected even higher rates of persistent hypercalciuria (3 of 4 patients with long-term follow-up), suggesting the need of ongoing monitoring in IIH. 

Our patients with malignancy-related hypercalcemia (n=4) demonstrated a treatment-resistant and repeated course. This was an expected finding as primary disease control was not achieved in these patients. Mild hypercalcemia not necessitating treatment with spontaneous resolution in the patient with FHH was compatible with the classically known clinical course of FHH (2).

### Study Limitations

This study has a few limitations. The most noteworthy limitation seems to be the small size of the study group, creating difficulty in comparisons between groups. Secondly, molecular analysis of CYP24A1 mutations, if performed, would have reinforced diagnosis of IIH. Thirdly, a longer follow-up period would have provided more detailed information about natural course of both hypercalcemia and hypercalciuria, recurrences, and complications such as nephrolithiasis.

## CONCLUSION

In conclusion, this study shows once again that childhood hypercalcemia may be due to a variety of factors. A considerable fraction of the patients remain asymptomatic, while many cases exhibit a non-specific and heterogeneous clinical presentation, resulting in delayed diagnosis. Mild cases may not be recognized. Moreover, symptoms may be missed in the cases with coexistence of a serious illness such as malignancy. On the other hand, serious complications may only be avoided with correct diagnosis and prompt intervention. Suspicion of hypercalcemia and measurement of serum calcium level in children with clinical findings non-attributable to any other clinical condition is essential.

## Figures and Tables

**Table 1:**
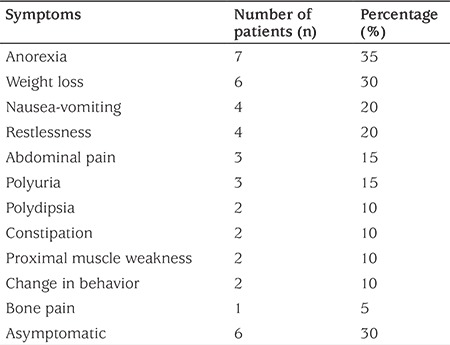
Frequency of symptoms related with hypercalcemia on admission

**Table 2:**
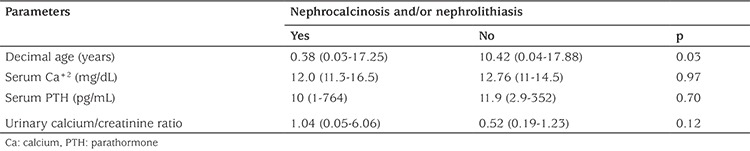
Comparison of different parameters based on presence of nephrocalcinosis and/or nephrolithiasis
